# Supplementary figures and images for: A R-Script for Generating Multiple Sclerosis Lesion Pattern Discrimination Plots
Source: Brain Sci. 2021 Jan 12;11(1):90. doi: 10.3390/brainsci11010090 (PMC7830121; doi:10.3390/brainsci11010090)

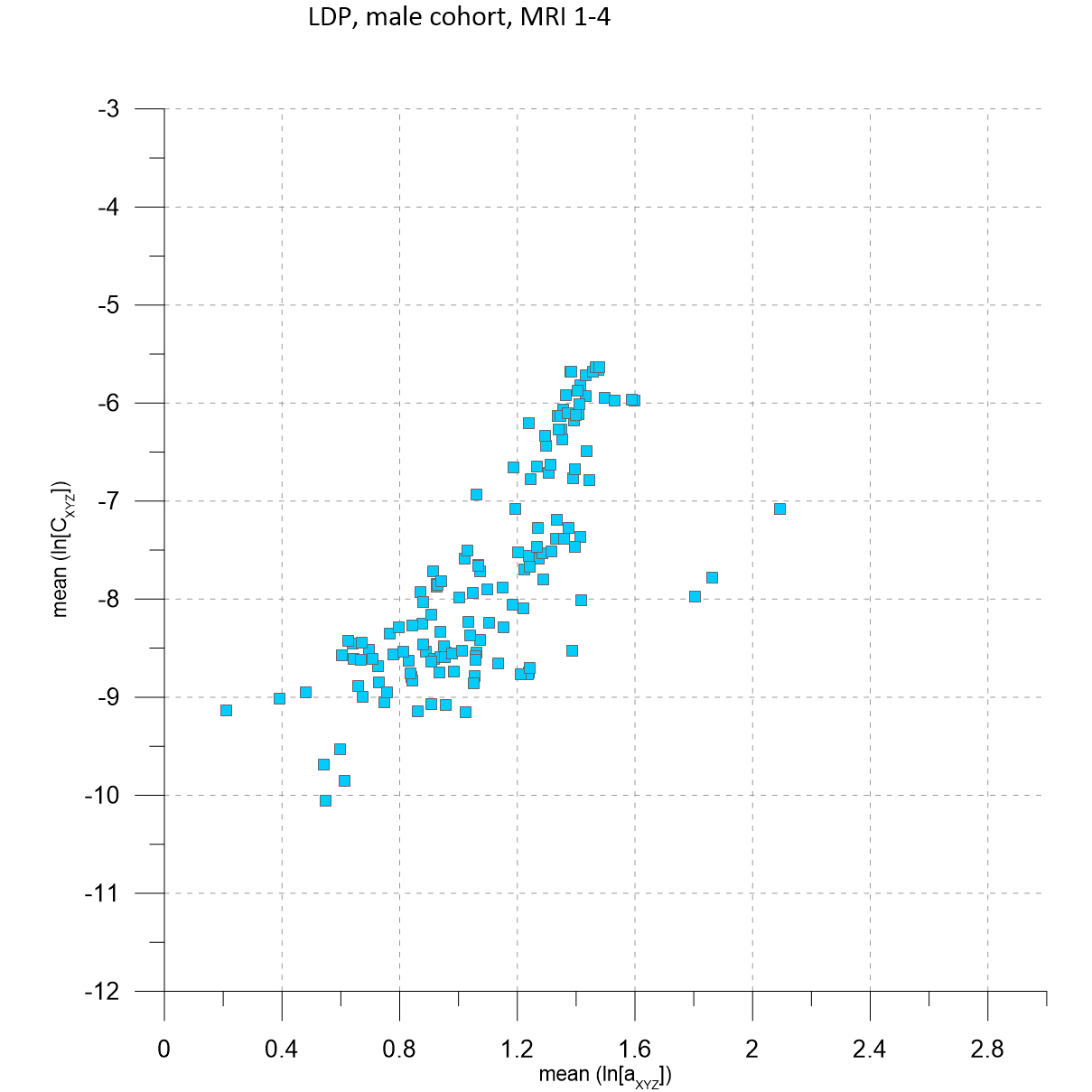

Supplement: Supplementary file 1 [file brainsci-11-00090-s001.zip › LDP_Supplement.jpg]

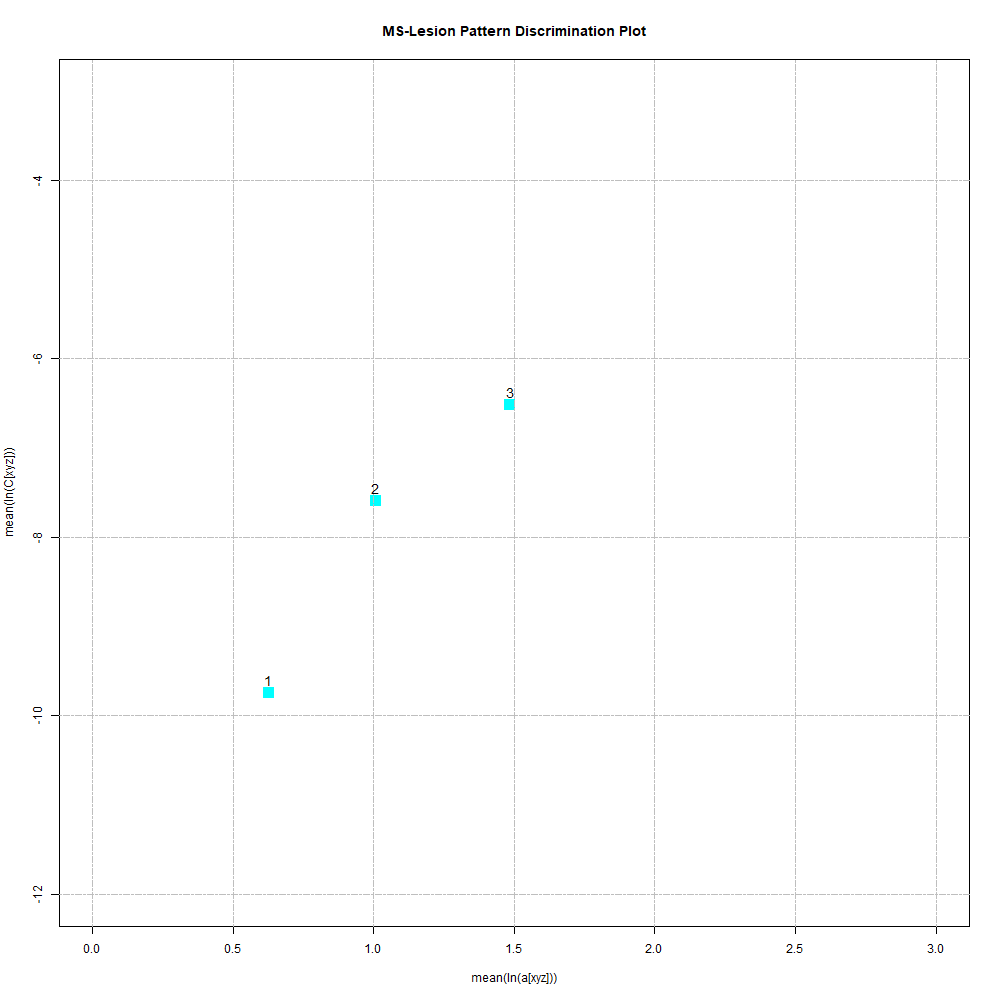

Supplement: Supplementary file 1 [file brainsci-11-00090-s001.zip › MNI_LDP.png]

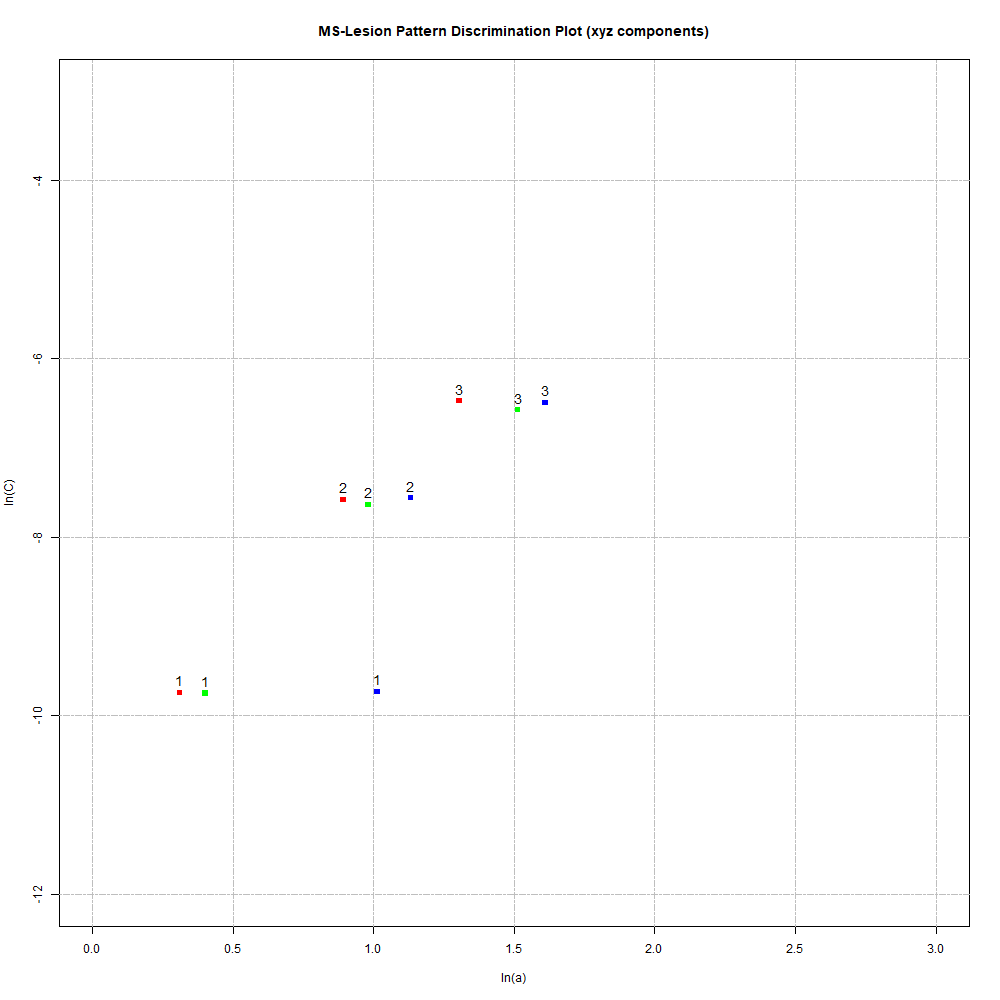

Supplement: Supplementary file 1 [file brainsci-11-00090-s001.zip › MNI_LDP_xyz.png]

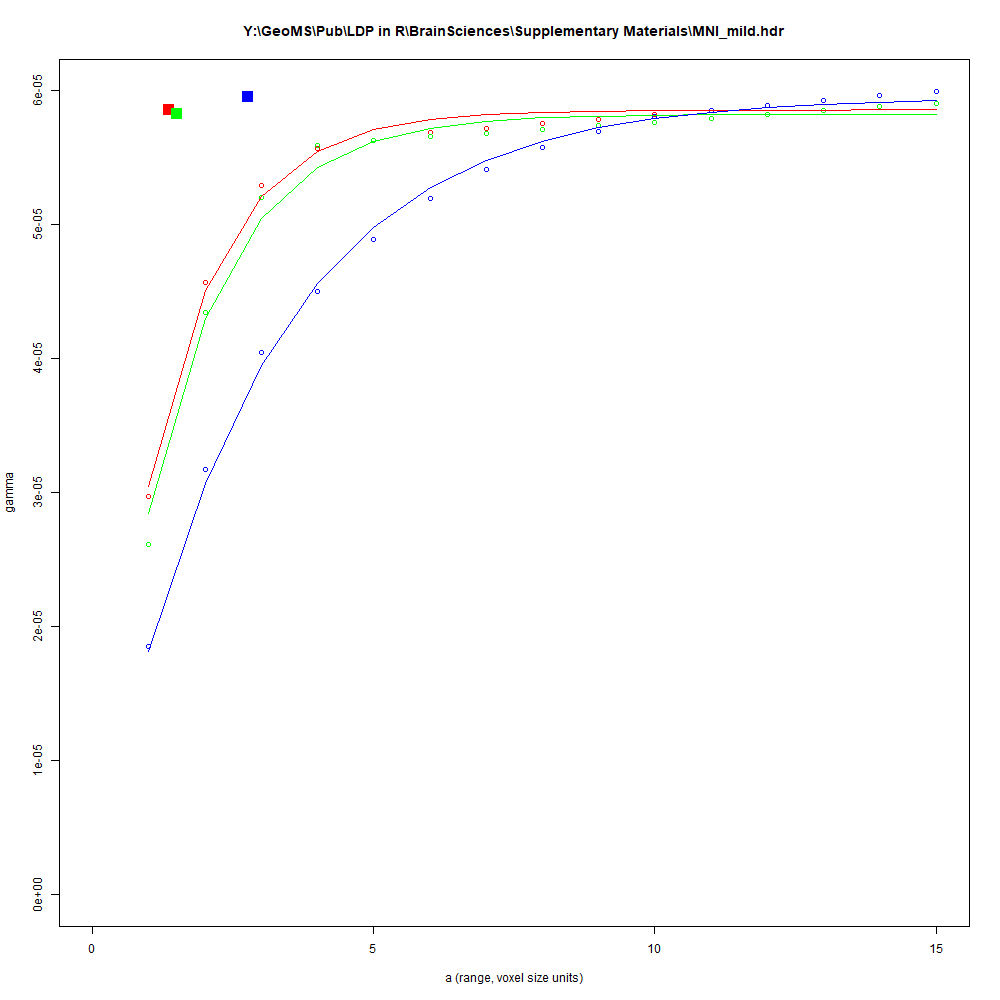

Supplement: Supplementary file 1 [file brainsci-11-00090-s001.zip › MNI_mild.hdr_variograms.png]

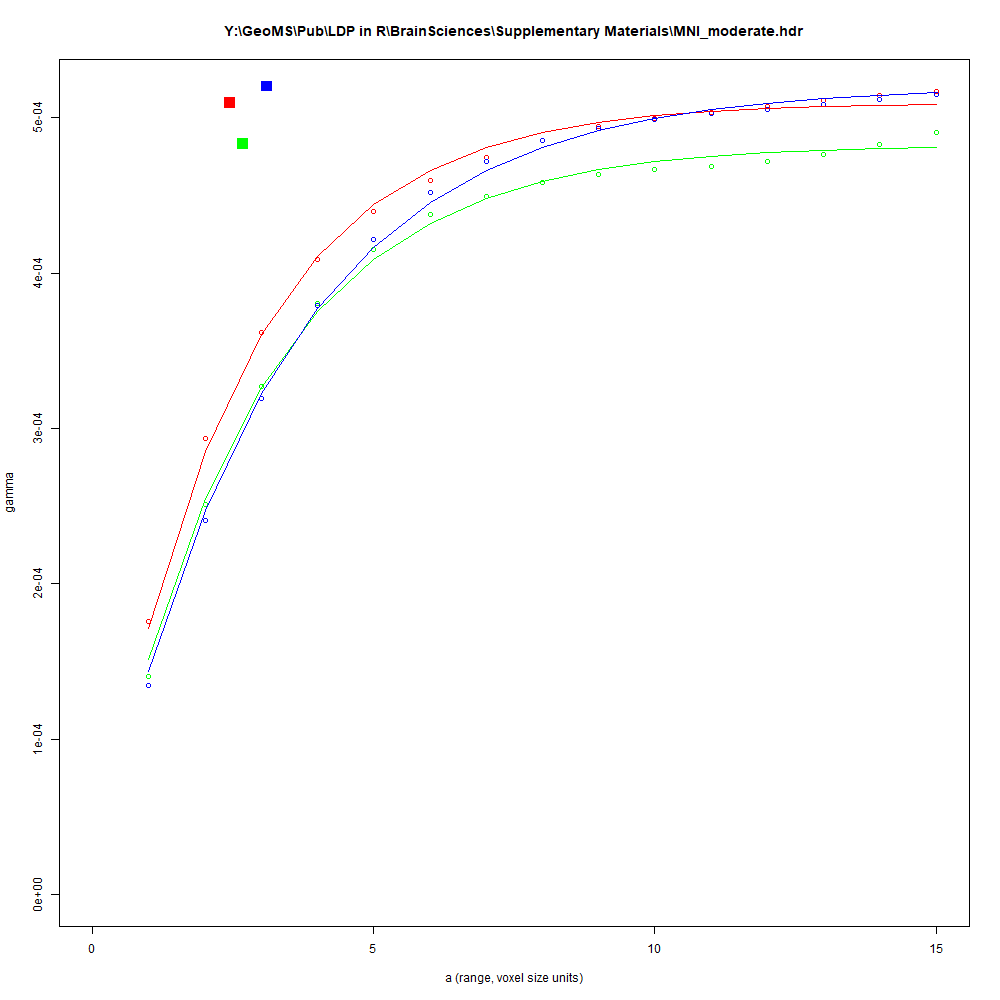

Supplement: Supplementary file 1 [file brainsci-11-00090-s001.zip › MNI_moderate.hdr_variograms.png]

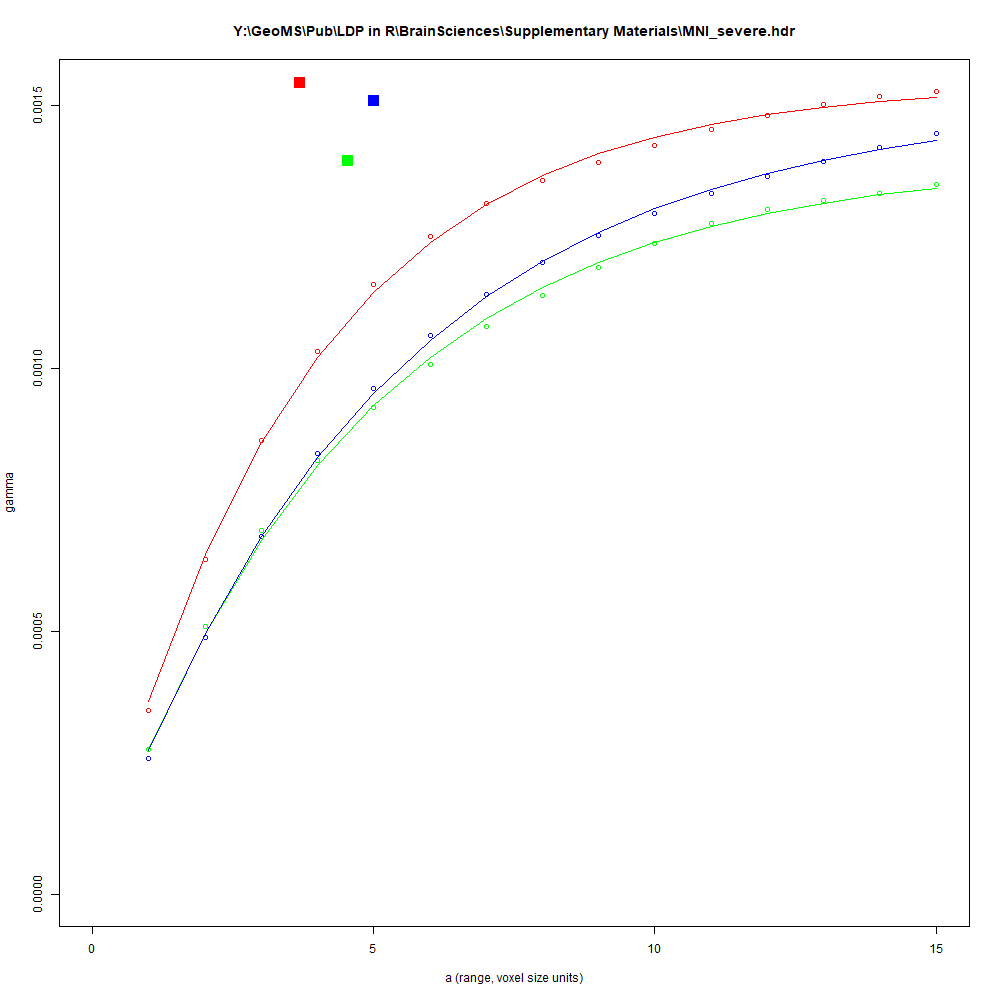

Supplement: Supplementary file 1 [file brainsci-11-00090-s001.zip › MNI_severe.hdr_variograms.png]
